# Health Outcomes of Kansas City’s Vulnerable Patients Following Shutdown: An Assessment of Blood Pressure Among Sojourner Health Clinic Patients

**DOI:** 10.7759/cureus.29057

**Published:** 2022-09-11

**Authors:** Fahad Qureshi, Kevin Varghese, Kashif Javid, Srivats Narayanan, Edwin Kraemer

**Affiliations:** 1 School of Medicine, University of Missouri at Kansas City, Kansas City, USA; 2 Department of Family Medicine, University Health Truman Medical Centers, Kansas City, USA

**Keywords:** student-run free clinic, care, access, homelessness, high blood pressure

## Abstract

Background

In downtown Kansas City, patients who face homelessness or unstable housing situations may have been negatively affected by the shutdown of Sojourner Health Clinic (SOJO), a free student-run clinic that provides primary care predominantly to these patients. Research shows that blood pressure (BP) increases within weeks or months of interruption of antihypertensive therapy, especially in patients with advanced age and polypharmacy. Therefore, this study will examine how patients’ blood pressure changed after the closure of Sojourner Health Clinic.

Methods

The study population consists of Sojourner Health Clinic patients who were seen both before March 2020 (shutdown) and during/after July 2020 (clinic reopening). Participants are selected at random. No additional data is collected outside of routine treatment for this institutional review board (IRB)-exempt project. A study coordinator reviews charts via Sojourner electronic medical record (EMR) and collects the latest BP available before March 2020 and the first BP available during/after July 2020. No identifying information is collected. The mean systolic pressures, mean diastolic pressures, and mean arterial pressures (MAP) are compared via paired t-test for statistical significance.

Results

There was a statistically significant decrease in patients’ MAP and diastolic BP after the closure of the clinic. However, there was not a statistically significant change found in patients’ systolic BP. The clinical significance of these results is limited by the minimal magnitude of change.

Conclusions

These findings run counter to our expectations since we believed that the closure of Sojourner Health Clinic would correlate with worsened markers of health, such as blood pressure control. It may be possible that the sampled patients turned to other sources for health maintenance and antihypertensive therapy during clinic closure. Future studies could explore these possibilities.

## Introduction

In urban African American populations, hypertensive patients have decreased physical functioning ability, decreased general health, and other comorbidities when compared to normotensive patients [1]. Patients with lower socioeconomic statuses (SES) are at increased risk for chronic medical conditions including hypertension (HTN), asthma, and obesity, preexisting conditions that increase the risk of severe COVID-19 [2]. The COVID-19 pandemic has also exacerbated health inequities by threatening employment, housing security, and healthcare access [3].

In downtown Kansas City, patients who face homelessness or unstable housing situations may have been negatively affected by the shutdown of Sojourner Health Clinic (SOJO), a free student-run clinic that provides primary care predominantly to these patients [4]. Many of the patients at Sojourner Health Clinic are aging, African American, and/or homeless. Uninsured and underserved patients receive medical care and medications free of cost at this weekly clinic. Patients are generally given a 28-day supply of medication. Sojourner Health Clinic (SOJO) is staffed by a multidisciplinary team of medical, pharmacy, physician assistant, and social worker students, as well as resident physicians and attending physicians.

SOJO was closed in March 2020 due to the COVID-19 pandemic. During the following months, no patients were seen, nor medication dispensed. It was not until July 2020 that the clinic reopened for all patients, as per the guidelines of the Centers for Disease Control and Prevention and the affiliate University of Missouri-Kansas City (UMKC) School of Medicine. Although SOJO made efforts to advertise this reopening to the urban Kansas City population, the initial weeks of July 2020 showed much lower patient numbers on a weekly basis. It took multiple weeks to months for some established patients to return to SOJO.

As mentioned above, the patients of the Sojourner Health Clinic were not seen for at least four months; in most cases, it took far more months for patients to be seen again in the clinic and receive medications. Research shows that blood pressure (BP) increases within weeks or months of interruption of antihypertensive therapy, especially in patients with increased age, higher BP with medication, and polypharmacy [5,6]. This could be true of our SOJO patient population, which tends to be elderly, African American, and/or homeless.

We analyzed how returning patients’ blood pressure changed after the closure of Sojourner Health Clinic. We hypothesized that since SOJO patients were not able to be seen for multiple months, their biomarkers such as BP worsened during the pandemic. We compared the SOJO patients’ systolic BP, diastolic BP, and mean arterial pressure (MAP) before and after the closure of Sojourner Health Clinic.

This data is important for us to explore, as it would elucidate how our patient population’s BP changed during the pandemic. We can also stratify the data to determine whether a certain age group, race, or housing status was disproportionately affected.

## Materials and methods

Drafting and approvals

Sojourner Health Clinic medical students drafted the general outline of this project and presented it for faculty approval in September 2020. Because BP is routinely recorded for every patient at every visit, no additional interventions or changes were made outside of routine care. This was a completely anonymous and randomized study. No patient identifiers were collected outside basic demographics. Randomization was provided by the fact that patients were only made eligible if they attended the SOJO clinic (i.e., randomization by participation). The student investigators then applied for and received an exemption from the University of Missouri-Kansas City (UMKC) School of Medicine Institutional Review Board (IRB). This study was conducted in accordance with the principles outlined in the Helsinki Declaration.

Selection and description of participants

Sojourner Health Clinic has a long-established population of patients. Patients are typically residents of urban Kansas City, oftentimes homeless or staying in shelters. The patient population is aging and mostly African American. However, adults of all ages, races, and housing statuses are represented in the SOJO patient population.

Patients who were seen at Sojourner Health Clinic both before March 15, 2020, and after July 1, 2020, were considered eligible for participation in this study. This cohort of patients would have been seen both before the SOJO pandemic shutdown and after clinic reopening. Furthermore, the patients must have a diagnosis of hypertension and be currently receiving antihypertensive treatment from SOJO. Lastly, eligible participants in this study must have had recorded BP readings for both visits in the medical record. Because the same patient was compared to themself at a later point in time, each patient served as their own control. Calculating BP change as opposed to absolute values allowed us to minimize confounding.

There were no specific inclusion criteria regarding the severity of HTN, the number of antihypertensives that the patient was prescribed, or specific comorbidities. Because of the low number of patients visiting SOJO after reopening, the study designers sought to maximize participants.

One exclusion criterion for this study during conduction was the type of patient seen in July 2020. When SOJO reopened, the clinic had a policy of screening potential patients for COVID-19 symptoms such as fever, cough, cold, sick contact, or recent travel. If a patient screened positive, then they were not seen in the clinic. In this manner, some patients who may have been eligible for this study were not seen in the clinic and thereby excluded. Furthermore, the SOJO clinic patient numbers after clinic reopening were far lower than the patient volume prior to shutdown. Because of this, many patients who were eligible were not yet seen in the clinic after reopening at the time of data collection.

Data collection

After IRB exemption and faculty mentor approval of the project, student investigators began data collection. SOJO utilizes an electronic medical record (EMR) named “Osler,” where patient charts are stored. For every week after July 1, 2020, student investigators screened patients who attended the clinic for the inclusion criteria listed above. As mentioned, there were no changes to the patient’s routine visit for the conduction of this study.

If the patient met the listed criteria, then the student investigator accessed the last note before July 1, 2020. Using a standardized questionnaire (Appendix), the student investigator collected information regarding gender, age, race, and housing status. Lastly, the student investigator opened the last clinic encounter note available before July 1, 2020, and noted the systolic BP in millimeters of mercury (mmHg) and the diastolic BP in millimeters of mercury. After collecting this information, the student investigator opened the first clinic encounter note available after July 1, 2020, and noted the systolic BP in millimeters of mercury and the diastolic BP in millimeters of mercury. No other information other than the parameters listed above was collected. Data collection occurred between November 2020 and February 2022.

Student investigators were provided a Google Forms (Google, Inc., Mountain View, CA, USA) link to use for data collection. Investigators filled the form with the information from the patient chart, selecting options when appropriate or inputting the BP in millimeters of mercury when appropriate. After submitting the form for an individual patient, the data populated Google Sheets that provided the data in a tabular form for comparison and analysis. Duplicates were checked for flagging identical repeated data in the Google Sheets, which did not occur.

Statistical analysis

When data collection ended in February 2022, the student investigators performed statistical analysis using the amassed data in the populated Google Sheets. First, the investigators analyzed the demographics of the study participants, categorizing the data by gender, race, age, and housing status. Basic descriptive functions within Google Sheets allowed for determining which were the predominant demographic characteristics represented in the sample population. Next, the investigators determined what was the mean systolic pressure before the shutdown, the mean diastolic pressure before the shutdown, the mean systolic pressure after the shutdown, and the mean diastolic pressure after the shutdown, with confidence intervals based on sample size and mean deviation. The investigators also calculated the mean arterial pressure for each patient both before and after shutdown. This was achieved using the formula cited for calculating the mean arterial pressure (mean arterial pressure = 2/3 × diastolic BP + 1/3 × Systolic BP) [7].

Next, the student investigators completed the analysis via Microsoft Excel (Microsoft Corp., Redmond, WA, USA). Excel contains functionality to perform analyses such as Student’s t-test. Student’s t-test is an analytic method that determines whether the difference between the means of two samples is statistically significant [8]. In this case, because the investigators compared the same group to itself at a later point in time, the ideal test is a paired t-test [9,10]. Using the “t-test” functionality, the student investigators made comparisons of the mean systolic pressure, mean diastolic pressure, and mean arterial pressure when they were grouped according to “before pandemic” and “after pandemic.” The p-value for statistical significance was set at 0.025 to allow for a one-tailed test without the need for prediction about the direction of effect.

## Results

Sixty-one patients were deemed eligible to participate in this study, and data were collected from this population. There were no duplicate patient entries. The major gender represented was male, at 68.85%. Most of the patients fell between the ages of 40 and 69 years old, making up 91.80% of the sample. There were no patients younger than 30 years old nor older than 79 years old in our study. The most common race was black, at 34.43%. The most common housing status was homeless, at 45.90%. Data on the aforementioned patient demographic information are displayed in Table 1.

**Table 1:** Eligible patient demographic information

Patient demographic		Number (%)
Gender	Male	42 (68.85)
	Female	19 (31.15)
Age	30-39	4 (6.56)
	40-49	18 (29.51)
	50-59	23 (37.70)
	60-69	15 (25.49)
	70-79	1 (1.64)
Race	Black/African American	21 (34.43)
	White/European	18 (29.51)
	Hispanic/Latino	14 (22.95)
	Asian American	3 (4.92)
	Other	5 (8.20)
Housing status	Homeless	28 (45.90)
	Rent or own housing	23 (37.70)
	Other	10 (16.39)
Total		61 (100)

We saw that the change in diastolic blood pressure showed variety from patient to patient. Every measure of BP decreased after the SOJO shutdown for this sample of patients. With a level of significance set at p = 0.025, two changes were found to be statistically significant, and one change was found not to be statistically significant. The decrease in systolic BP was not statistically significant. Statistical information regarding the change in systolic blood pressure is illustrated in Table 2.

**Table 2:** Change in systolic blood pressure utilizing Student’s t-test

		Before shutdown	After shutdown
Mean (standard deviation)		134.67 (13.67)	131.85 (17.53)
Pearson correlation	0.06002		
P(T<=t) one-tail	0.15796		

We find that the 3.76 mmHg decline in diastolic BP is statistically significant at p = 0.02. Statistical information regarding the change in diastolic blood pressure is illustrated in Table 3.

**Table 3:** Change in diastolic blood pressure utilizing Student’s t-test

		Before shutdown	After shutdown
Mean (standard deviation)		84.89 (13.65)	81.13 (11.43)
Pearson correlation	0.41609		
P(T<=t) one-tail	0.01807		

We find that the 3.59 mmHg decline in mean arterial pressure is statistically significant at p = 0.01. Statistical information regarding the change in mean arterial blood pressure is illustrated in Table 4.

**Table 4:** Change in mean arterial blood pressure utilizing Student’s t-test

		Before shutdown	After shutdown
Mean (standard deviation)		101.51 (10.65)	97.92 (10.61)
Pearson correlation	0.35731		
P(T<=t) one-tail	0.01168		

## Discussion

The results of this study are significant because of the role different measures of BP play in predicting overall morbidity and mortality. Existing literature has found that systolic and diastolic blood pressures are predictors of important outcomes. These outcomes include thrombotic stroke, myocardial infarction, and death [11]. Mean arterial pressure has been shown to be a strong predictor of all-cause and cardiovascular disease mortality in the middle-aged population and elderly [12]. Therefore, declines in these measures are significant as they may indicate improvements in overall health and increase predicted life expectancy.

These findings run counter to our expectations since we hypothesized that lack of access to our clinic would correlate with worsened markers of health. Patients received neither medical care nor medications from SOJO during the closure. SOJO patients have identified the clinic as their primary source of care in previous surveys. We traditionally prescribe a 28-day supply of medications, so it is likely that patients ran out of SOJO medications during closure.

It may be possible that the sampled patients turned to other sources for health maintenance and antihypertensive therapy during clinic closure. It has been shown that the COVID-19 pandemic has widened health inequity across multiple measures [13]. Other possible reasons for these findings include changes in diet during the pandemic, more time for self-care, the expansion of Medicare/Medicaid, or the utilization of stimulus funds for healthcare.

Our findings run counter to the literature, which indicates that the COVID-19 pandemic has generally resulted in worsened measures of health [2,3,13]. One way our study contributes to the literature is by bringing complexity to this trend. We also open discussion on the possibility of patients turning to alternative means of healthcare when their traditional sources were interrupted.

There are a few limitations to our study. The first limitation is our sample size of 61 patients. The sample size is enough for the minimum generally considered acceptable to represent the population and is in line with the literature, although more participants would benefit our analysis [14-16]. Another limitation is that SOJO was not seeing patients who were exhibiting COVID-19 symptoms at the time of data collection, which may have been a form of selection bias via exclusion [17]. Lastly, although the collection of BP was standardized using trained personnel, self-control, and the same equipment, there may have been slight unpreventable differences in measurement [18-20]. Finally, this was a wholly non-interventional, retrospective review of medical records.

## Conclusions

In summary, the results of this study indicate that the sampled SOJO patients experienced a significant decline in their diastolic BP after the closure of the clinic for roughly four months. Furthermore, there was a significant decline in MAP. The clinic is roughly at 70% of the patient volume regularly seen before July 2020, averaging seven patients per week in comparison to 10 patients per week.

Although there are possible confounding factors as we discussed, we are able to see a trend unlike one otherwise reported in the literature. We conducted this observational analysis of routine data collected in the clinic so that we could determine whether there were changes in BP between two time points. Upon finding significant changes, we would then like to conduct a second study exploring possible reasons. We feel that this information is valuable to the free clinic community because it encourages other clinics to consider similar questions for their own patients as we move toward a “new normal.”

Future studies could first explore the potential alternative sources of healthcare that SOJO patients turned to during the pandemic closure. We would like to investigate this via a questionnaire or survey. Identifying community partners and collaborating across organizations may better serve our patients. We encourage other student-run free clinics to complete similar analyses to contribute to the literature on the effect of clinic closure on markers of health.

## Appendices

Table 5 shows the standardized questionnaire used to collect information regarding gender, age, race, and housing status.

**Table 5:** Standardized questionnaire

Demographic variables	
1. Gender	a. Female
	b. Male
	c. Other
2. Race	a. Black/African American
	b. White/European
	c. Hispanic/Latino
	d. Asian American
	e. Other
3. Age	a. 0-9
	b. 10-19
	c. 20-29
	d. 30-39
	e. 40-49
	f. 50-59
	g. 60-69
	h. 70-79
	i. 80-89
	j. 90-99
4. Housing status	a. Homeless (shelter or on streets)
	b. Rent or own housing
	c. Other
5. Last systolic BP available before July 1, 2020	
6. Last diastolic BP available before July 1, 2020	
7. First systolic BP available after July 1, 2020	
8. First diastolic BP available after July 1, 2020	
